# Lead Exposure during Early Human Development and DNA Methylation of Imprinted Gene Regulatory Elements in Adulthood

**DOI:** 10.1289/ehp.1408577

**Published:** 2015-06-26

**Authors:** Yue Li, Changchun Xie, Susan K. Murphy, David Skaar, Monica Nye, Adriana C. Vidal, Kim M. Cecil, Kim N. Dietrich, Alvaro Puga, Randy L. Jirtle, Cathrine Hoyo

**Affiliations:** 1 Department of Community and Family Medicine, and; 2 Department of Obstetrics and Gynecology, Duke University Medical Center, Durham, North Carolina, USA; 3 Division of Epidemiology and Biostatistics, Department of Environmental Health, Center for Clinical and Translational Science and Training, University of Cincinnati (UC), Cincinnati, Ohio, USA; 4 Department of Biological Sciences, Center for Human Health and the Environment, North Carolina State University (NCSU), Raleigh, North Carolina, USA; 5 Department of Epidemiology, University of North Carolina at Chapel Hill, Chapel Hill, North Carolina, USA; 6 Cincinnati Children’s Environmental Health Center, Cincinnati Children’s Hospital Medical Center, UC College of Medicine, Cincinnati, Ohio, USA; 7 Department of Radiology,; 8 Department of Pediatrics,; 9 Department of Environmental Health,; 10 Center for Environmental Genetics, and; 11 Division of Epidemiology and Biostatistics, UC College of Medicine, Cincinnati, Ohio, USA; 12 Department of Oncology, McArdle Laboratory for Cancer Research, University of Wisconsin-Madison, Madison, Wisconsin, USA; 13 Department of Sport and Exercise Sciences, Institute of Sport and Physical Activity Research, University of Bedfordshire, Bedford, Bedfordshire, United Kingdom

## Abstract

**Background:**

Lead exposure during early development causes neurodevelopmental disorders by unknown mechanisms. Epidemiologic studies have focused recently on determining associations between lead exposure and global DNA methylation; however, such approaches preclude the identification of loci that may alter human disease risk.

**Objectives:**

The objective of this study was to determine whether maternal, postnatal, and early childhood lead exposure can alter the differentially methylated regions (DMRs) that control the monoallelic expression of imprinted genes involved in metabolism, growth, and development.

**Methods:**

Questionnaire data and serial blood lead levels were obtained from 105 participants (64 females, 41 males) of the Cincinnati Lead Study from birth to 78 months. When participants were adults, we used Sequenom EpiTYPER assays to test peripheral blood DNA to quantify CpG methylation in peripheral blood leukocytes at DMRs of 22 human imprinted genes. Statistical analyses were conducted using linear regression.

**Results:**

Mean blood lead concentration from birth to 78 months was associated with a significant decrease in PEG3 DMR methylation (β = –0.0014; 95% CI: –0.0023, –0.0005, p = 0.002), stronger in males (β = –0.0024; 95% CI: –0.0038, –0.0009, p = 0.003) than in females (β = –0.0009; 95% CI: –0.0020, 0.0003, p = 0.1). Elevated mean childhood blood lead concentration was also associated with a significant decrease in IGF2/H19 (β = –0.0013; 95% CI: –0.0023, –0.0003, p = 0.01) DMR methylation, but primarily in females, (β = –0.0017; 95% CI: –0.0029, –0.0006, p = 0.005) rather than in males, (β = –0.0004; 95% CI: –0.0023, 0.0015, p = 0.7). Elevated blood lead concentration during the neonatal period was associated with higher PLAGL1/HYMAI DMR methylation regardless of sex (β = 0.0075; 95% CI: 0.0018, 0.0132, p = 0.01). The magnitude of associations between cumulative lead exposure and CpG methylation remained unaltered from 30 to 78 months.

**Conclusions:**

Our findings provide evidence that early childhood lead exposure results in sex-dependent and gene-specific DNA methylation differences in the DMRs of PEG3, IGF2/H19, and PLAGL1/HYMAI in adulthood.

**Citation:**

Li Y, Xie C, Murphy SK, Skaar D, Nye M, Vidal AC, Cecil KM, Dietrich KN, Puga A, Jirtle RL, Hoyo C. 2016. Lead exposure during early human development and DNA methylation of imprinted gene regulatory elements in adulthood. Environ Health Perspect 124:666–673; http://dx.doi.org/10.1289/ehp.1408577

## Introduction

Elevated lead exposure in early life is associated with growth retardation, neurotoxicity, impaired cognitive development in infancy, and deficits in attention and executive function (Bellinger et al. 1986, 1987). Accumulating evidence also indicates that the effect of lead exposure in early childhood can change neurochemistry (Binns et al. 2007), cause neurobehavioral and cognitive deficits in later life (Finkelstein et al. 1998; Sanders et al. 2009; Winneke et al. 1983; Zahran et al. 2009), decrease brain volume, and increase the rate of criminal arrest in adulthood (Cecil et al. 2008; Wright et al. 2008). Although the current actionable concentration for lead is 5 μg/dL in the United States, the threshold for its toxicity is unknown. Furthermore, the mechanisms by which lead exposure affects diverse neuropathological outcomes is not clearly defined, although epigenetic mechanisms have been proposed (Wright et al. 2010).

Environmental exposures to both physical and chemical agents, especially during early development, can induce alterations in DNA methylation that alter disease susceptibility in adulthood (Bernal et al. 2013; Dolinoy et al. 2006, 2007; Waterland and Jirtle 2003). Animal studies likewise suggest that epigenetic modifications may link lead exposure to neurotoxicity and attention deficit disorders (Faulk et al. 2013; Luo et al. 2014), but stable epigenetic targets responsive to early lead exposure in humans remain uncharacterized.

Because of the relative ease of measuring genomic DNA cytosine methylation at CpG dinucleotides, this end point is the most commonly investigated epigenetic modification in epidemiologic studies. *In vitro* and *in vivo* studies demonstrate that DNA methylation is altered by exposure to toxic metals, including arsenic, cadmium, and lead (Bolin et al. 2006; Reichard et al. 2007; Takiguchi et al. 2003). The only epigenetic studies conducted in humans thus far have evaluated DNA methylation at *Alu* and *LINE* repeat elements. They showed associations between maternal patella lead levels and global DNA hypomethylation in newborns (Pilsner et al. 2009) and adult males (Wright et al. 2010). Although the biological significance of reduced DNA methylation at repetitive elements in lead-exposed humans is unknown, this epigenetic change also occurs frequently in cancer, where it is believed to result in chromosomal instability and genomic mutations (Wilson et al. 2007).

Imprinted genes are characterized by parent-of-origin–dependent monoallelic expression, with the functionally haploid state controlled by differentially methylated regions (DMRs). The inherited imprint methylation marks at these DMRs are established during gametogenesis (i.e., gametic imprints) or early in embryogenesis (i.e., somatic imprints) (Barlow 2011; Reik and Walter 2001). Epigenetic dysregulation of imprinted genes is associated with diseases, including cancer, diabetes, obesity, and developmental and neurological disorders (Ishida and Moore 2013; Murphy and Jirtle 2003). DNA methylation marks at imprinted DMRs are generally maintained in tissues from the three germ layers (Murphy et al. 2012b; Waterland et al. 2010; Woodfine et al. 2011). Furthermore, with imprinted genes often occurring in clusters (Edwards et al. 2007) and with the potential for network regulation (Varrault et al. 2006), the methylation status of a single DMR could affect the expression of multiple genes.

The availability of childhood blood lead concentration data and adult peripheral blood DNA from the Cincinnati Lead Study cohort (Cecil et al. 2008; Dietrich et al. 1987, 1993, 2001) provided the impetus for the first determination of prenatal and postnatal lead exposure associations with DNA methylation in adulthood for the DMRs of 22 imprinted genes, as recently described (Skaar et al. 2012).

## Materials and Methods

*Study population*. Participants comprised 41 men and 64 women born between 1979 and 1984 who were enrolled in the Cincinnati Lead Study and were successfully recontacted in 2008–2010. Accrual and lead measurement methods have been described in detail (Cecil et al. 2008; Dietrich et al. 1987). Briefly, pregnant women living in neighborhoods with high prevalence of pediatric lead poisoning were eligible. Women with type 1 or type 2 diabetes and neurological, psychiatric, or drug addiction disorders were excluded, as were offspring with defects or birth weight < 1,500 g. Using anodic stripping voltammetry (Roda et al. 1988), lead concentrations were measured in first- and second-trimester maternal peripheral blood and in blood collected from their children at 10 days of age, every 3 months for the first 60 months, and every 6 months from 60 to 78 months. Umbilical cord blood was not measured due to clotting. During pregnancy, questionnaires were used to collect data on maternal race/ethnicity (black/white), the number of cigarettes smoked per day, education, occupation, continuous IQ, use of alcohol (yes/no), marijuana (yes/no), and narcotics (yes/no), and children’s IQ at age 6.5 years. The protocol was approved by review boards for Cincinnati, Duke, and North Carolina State Universities.

*Specimen handling.* During the 2008 visit, 108 participants with an average age of 27 years (25–30 years) provided informed consent before peripheral blood specimens for DNA methylation analysis were collected; 105 also had covariable data. Blood was collected in K_2_EDTA-treated vaccutainer tubes, centrifuged for plasma and buffy coat isolation, and shipped to the Jirtle laboratory at Duke University for DNA methylation analysis.

*Quantitative methylation analysis.* DNA was extracted using Puregene reagents according to manufacturer’s protocol (Qiagen, Valencia, CA). In the Supplemental Material, Table S1 summarizes DMR amplicon cleavage fragments, including CpG content and positions. DNA amplification used a touchdown polymerase chain reaction (PCR) protocol.

Quantitative DNA methylation analysis was performed in two batches using the Sequenom MassARRAY EpiTYPER (Sequenom, San Diego, CA). Primers for human imprinted genes were designed with the use of Epidesigner software (Sequenom) to amplify approximately 400–600 bp of the imprinted gene DMRs. Cycling conditions for touchdown PCR used are in Supplemental Material, Table S2, and primers and amplicon data are provided in Supplemental Material, Table S3. Genomic DNA (2 μg) was treated with sodium bisulfite using the EpiTect kit, according to manufacturer’s protocols (Qiagen, Valencia, CA). Bisulfite-converted DNA (50 ng) was amplified by PCR using HotStarTaq (Qiagen).

PCR products were processed by dephosphorylation of unincorporated dNTPs. They were then transcribed *in vitro* with concurrent RNase cleavage using T-cleavage assays according to the manufacturer’s standard protocol (Sequenom). The transcription reaction was conditioned to remove cations by adding 20 μL H_2_O and 6 mg of Clean Resin (Sequenom). Subsequently, the samples were spotted on a 384-pad Spectro-CHIP (Sequenom), using a MassARRAY Nanodispenser (Samsung, Irvine, CA), followed by spectral acquisition on a MassARRAY analyzer compact MALDI-TOF MS (matrix-assisted laser desorption/ionization time-of-flight mass spectrometer) (Sequenom). The percent methylation of CpG sites for each cleavage fragment was determined using EpiTyper software (Sequenom).

The Sequenom MassARRAY measured DNA methylation using fragments of reverse-transcribed PCR products, and data output is in CpG “units” in which multiple CpG sites may reside within a single fragment produced by RNase cleavage of transcripts of DMR amplicons (see Supplemental Material, Table S1). The mass difference between fragments with “T” and “C” bases (bisulfite-converted unmethylated, and unconverted methylated cytosines, respectively) at CpG sites was detected, and the ratio of alternate masses was quantitated to generate the methylation fraction. For fragments with multiple CpGs, the methylation value was calculated as an average of all sites. Visual inspection of the output from the mass spectrometer for such fragments with multiple CpGs provided an estimate of the accuracy of this average for individual sites. The detection of primarily two peaks representing hypomethylated and hypermethylated fragments was indicative of strand-specific, *cis*-regulated differential methylation, and the methylation value for the entire fragment was considered to represent each individual CpG site. For most fragments with multiple CpG sites, this two-peak output was the case.

In the Supplemental Material, Table S1 summarizes fragments for each amplicon, CpG content, and position within the fragments and shows which fragments were excluded from analysis due to low or high mass outside the detection range, fragment duplication, overlap, or success rate below the 95% threshold. Methylation values for fragments with multiple CpG sites were weighted when included in DMR average methylation, and fragments with duplicate masses were included separately, such that the DMR average methylation is the same as if data for each CpG were available.

Reproducibility of 5–10% for MassARRAY methylation analysis was verified in triplicate for the 22 DMRs using control conceptus tissues representing the three germ layers. In these control fetal tissues, the mean DNA methylation ranged from 45% to 60%. In humans exposed to varying lead levels, the mean DNA methylation ranged from 37% to 74% (see Supplemental Material, Table S3). When > 5% of samples produced no signals, indicating errors in spotting or failures in amplification or cleavage, the run was discarded.

*Statistical analyses, DNA methylation, and covariable data.* A total of 172 CpG-containing cleavage fragments from 22 genomically imprinted gene DMRs had methylation percentages available for the 105 participants. The number of analyzed regions for each DMR and the mean methylation for each DMR computed from nonmissing CpG-containing fragments are in the last two columns of Supplemental Material, Table S3. The R-package was used for data analysis (R Core Team 2013).

Lead concentrations (micrograms per deciliter) were analyzed as age-specific concentrations cumulatively defined as the sum of blood lead measurements up to and including the value measured at that age, divided by the number summed (e.g., concentrations at 10 days + 3 months + 6 months/3 = average cumulative concentrations at age 6 months). Lead values were also arrayed and the maximum lead value for each participant was identified. Lead was also categorized into four developmental stages at measurement: prenatal (first- or second-trimester gestation); neonatal (age 10 days); early childhood, coinciding with higher concentrations characteristic of the crawling/oral exploratory developmental window (age 3–30 months); and middle childhood, coinciding with declining concentrations (age 33–78 months). Factors shown to be associated with lead concentrations, from previous analyses of these data (Cecil et al. 2008; Dietrich et al. 1987), and factors known to be associated with DNA methylation were evaluated for confounding in the overall mean and maximum saturated models. Only those with a *p*-value < 0.05 were retained in refined models. Factors evaluated for confounding were maternal education, smoking, and race, as well as offspring sex and batch. Only sex (male/female), batch (first or second), and smoking (none, ≤ 0.5, 1, 1.5, and 2 packs per day, computed from the number of cigarettes smoked daily, assuming 20 cigarettes in a pack) remained significant, and were retained in refined models.

Because some DNA methylation values were not normally distributed, the log_2_ of the standardized regression coefficients,


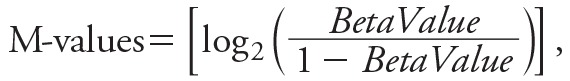


were used in adjusted linear regression models. These were compared with unstandardized regression coefficients, and the results were similar (data not shown). For ease of interpretation, we present unstandardized regression coefficients with 95% confidence intervals (CIs) in tables, whereas regression coefficients at each lead measurement are plotted without confidence intervals. A *p*-value ≤ 0.05 was considered statistically significant.

**Figure 1:**
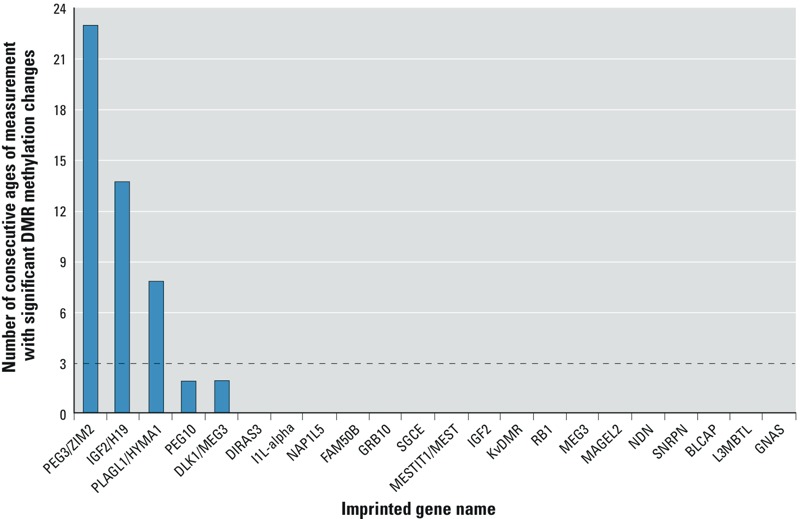
Number of consecutive ages of measurement with significant (*p* ≤ 0.05) association of DMR methylation with lead exposure from birth to age 78 months. The dashed line indicates the threshold for imprinted gene inclusion.

The limited sample size precluded adjustment for multiple comparisons. Instead, we included only DMRs for which the level of methylation of more than three CpG-containing fragments were correlated *r* > 0.8, suggesting *cis*-acting regulation (data not shown). The CpG-containing fragments also had to have persistent significant (*p* < 0.05) associations with lead exposure for any four consecutive mean lead measurements (e.g., associations were significant and in the same direction for lead levels measured at 3, 6, 9, and 12 months) as seen in Figure 1.

## Results

**Figure 2:**
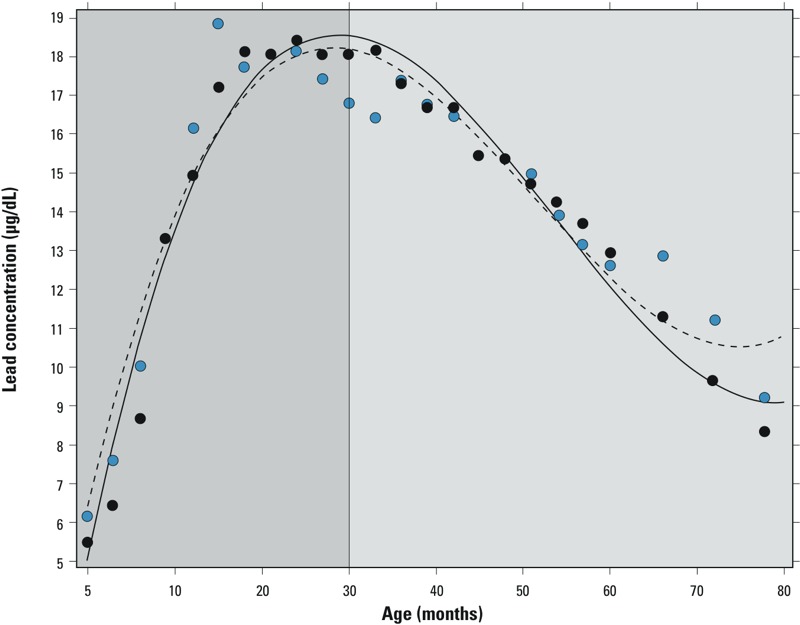
Mean postnatal circulating lead concentrations in males (blue circles, dashed line) and females (black circles, solid line) versus childhood age. Darker gray area indicates early childhood—10 days to 30 months; lighter gray area indicates middle childhood—30 to 78 months.)

*Study participants, lead concentrations, and DNA methylation.* The majority (90%) of participants were born to black women, ~ 70% had less than a high school education, and the median IQ was 75 (Table 1). Although ~ 50% of participants were exposed to cigarette smoke *in utero*, exposure to alcohol, marijuana, and narcotics was uncommon (< 13%). Prenatal mean (± SD) blood lead concentration was 9.1 ± 6.0 μg/dL, postnatal mean lead concentration was 13.6 ± 5.5 μg/dL, and the maximum mean was 25.3 ± 5.3 μg/dL. Blood lead concentrations in males and females were comparable throughout the study visits; the concentration increased rapidly with age, peaking at age 20–25 months, and then decreased (Figure 2). Lead concentrations and the distribution of covariable data were comparable with those who were successfully contacted again in 2008–2010 (*p* ≥ 0.14).

**Table 1:** Characteristics of study participants [*n* (%)].

Characteristic	Total sample	Males (*n*=41)	Females (*n*=64)
Race
White	10 (9.5)	5 (12.2)	5 (7.8)
Black	95 (90.5)	36 (87.8)	59 (92.2)
Education
≤High school	71 (67.7)	33 (80.5)	38 (59.3)
>High school	34 (32.4)	8 (19.5)	26 (40.6)
Range (years)	6–16	9–16	6–16
Maternal alcohol use
Yes	13 (12.4)	4 (9.8)	9 (14.1)
No	92 (87.6)	37 (90.2)	55 (85.9)
Maternal narcotic use
Yes	1 (1.5)	0 (0.0)	1 (1.6)
No	104 (98.5)	40 (100.0)	63 (98.4)
Maternal marijuana use
Yes	11 (10.5)	4 (9.8)	7 (10.9)
No	94 (89.5)	37 (90.2)	57 (89.1)
Maternal tobacco use during pregnancy^*a*^
None	48 (45.7)	23 (56.1)	25 (39.1)
<pack/day	43 (41.0)	13 (31.7)	30 (40.6)
1 pack/day	11 (10.5)	3 (7.2)	8 (12.5)
1.5 packs/day	2 (1.9)	1 (2.4)	1 (1.6)
2 packs/day	1 (1.0)	1 (2.4)	0 (0)
Birth weight (g) [median (range)]	3,096 (1,990–4,340)	3,184 (2,000–4,260)	3,040 (1,990–4,340)
Maternal IQ [median (range)]	75 (58–102)	74 (61–97)	76 (58–102)
Participant IQ [median (range)]	87 (50–116)	87 (50–111)	88 (67–116)
Lead concentrations during developmental windows (μg/L)
Childhood (birth to 78months) (mean)	13.6 (5.5)	13.7 (5.5)	13.5 (5.5)
Neonatal period (≤28 days)	14.5 (5.9)	14.5 (6.1)	14.5 (5.7)
Early childhood (age ≤30months)	14.5 (5.9)	14.5 (6.1)	14.5 (5.7)
Middle childhood (>30–78months)	13.0 (6.1)	13.2 (5.9)	12.8 (6.2)
Age (years) at blood draw for DNA methylation determination [median (range)]	26.7 (25.4–29.6)	26.9 (25.5–29.6)	26.5 (25.4–28.4)
^***a***^Packs per day is based on a typical American package of 20 cigarettes.			

Data for 37 of 172 CpG-containing fragments were missing for > 5% of participants, leaving 135 fragments for analysis. The mean DMR methylation levels for the 105 participants exposed to varying levels of lead ranged from 37% to 74% (see Supplemental Material, Table S3).

**Figure 3:**
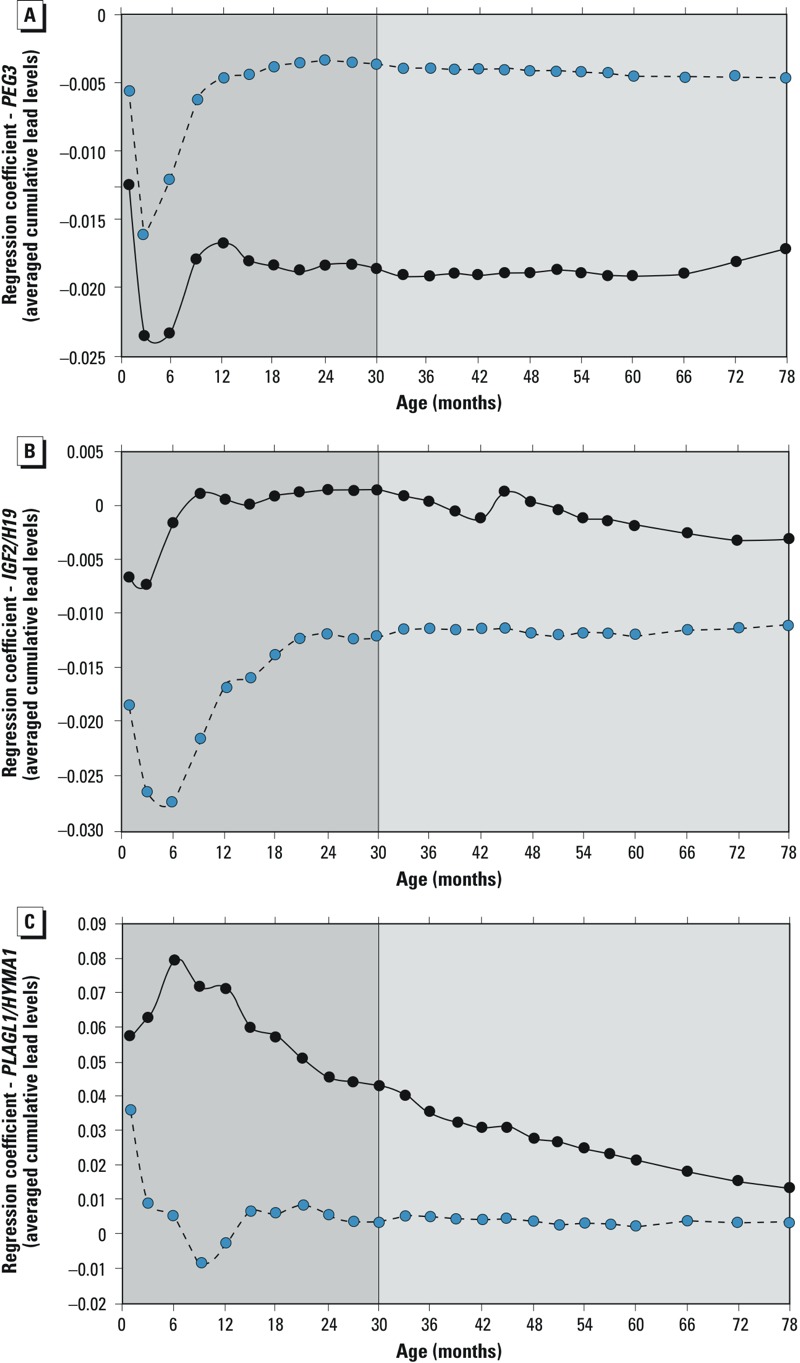
Unstandardized regression coefficients for associations between DMR methylation for *PEG3 *(*A*)*,* *IGF2/H19 *(*B*), and *HYMA/PLAGL1* (*C*) and the average cumulative lead level (obtained by summing up blood level measurements, up to and including the value measured at that age, divided by the number summed, in males (blue circles) and females (black circles). The darker gray area indicates early childhood; the lighter gray area indicates middle childhood.

*Associations between early lead exposure and DMR methylation.* Lead concentrations were highly correlated within individuals, especially in early childhood (see Supplemental Material, Table S4). Of the 22 DMRs, those for six imprinted genes had more than three CpGs with correlation > 0.8—*PEG10, DLK1/MEG3, DIRAS*, *PEG3*, *IGF2/H19,* and *PLAGL1/HYMA1.* However, the mean and maximum lead concentrations were persistently associated only with the DNA methylation of DMRs for *PEG3*, *IGF2/H19*, and *PLAGL1/HYMAI* (Table 2 and Figure 3; see also Supplemental Material, Table S5).

**Table 2:** Regression coefficients for the association between mean lead exposure and *PEG3, IGF2/H19*, and *PLAGL1/HYMAI* DMR methylation.*^a^* ^,^ *^b^*

DMR/CpG Lead exposure	Both sexes	Males	Females
	β (95% CI), *p*-value	β (95% CI), *p*-value	β (95% CI), *p*-value
*PEG3*
Mean life childhood lead levels (birth to age 78months)	–0.0014 (–0.0023, –0.0005), 0.002	–0.0024 (–0.0038, –0.0009), 0.003	–0.0009 (–0.0020, 0.0003), 0.1
Prenatal lead levels (2nd or 3rd trimester)	–0.0011 (–0.0028, 0.0005), 0.2	0.00001 (–0.0028, 0.0028), 1.0	–0.0017 (–0.0037, 0.0004), 0.1
Neonatal lead levels (age 10 days)	–0.0010 (–0.0025, 0.0004), 0.2	–0.0011 (–0.0030, 0.0007), 0.2	–0.0010 (–0.0035, 0.0016), 0.5
Early childhood levels (age 3–30months)	–0.0012 (–0.0020, –0.0004), 0.005	–0.0023 (–0.0036, –0.0009), 0.002	–0.0006 (–0.0016, 0.0005), 0.3
Middle childhood levels (age 33–78months)	–0.0013 (–0.0021, –0.0005), 0.002	–0.0018 (–0.0031, –0.0004), 0.02	–0.0009 (–0.0020, 0.0001), 0.1
*IGF2/H19*
Mean life childhood lead levels (birth to age 78months)	–0.0013 (–0.0023, –0.0003), 0.01	–0.0004 (–0.0023, 0.0015), 0.7	–0.0017 (–0.0029, –0.0006), 0.005
Prenatal lead levels (2nd or 3rd trimester)	–0.0004 (–0.0022, 0.0014), 0.7	0.0007 (–0.0025, 0.0040), 0.7	–0.0011 (–0.0033, 0.0011), 0.3
Neonatal lead levels (age 10 days)	–0.0013 (–0.0029, 0.0003), 0.1	–0.0009 (–0.0032, 0.0013), 0.4	–0.0025 (–0.0052, 0.0002), 0.08
Early childhood levels (age 3–30months)	–0.0009 (–0.0019, 0.00001), 0.06	0.0004 (–0.0014, 0.0022), 0.7	–0.0016 (–0.0027, –0.0005), 0.007
Middle childhood levels (age 33–78months)	–0.0013 (–0.0022, –0.0004), 0.005	–0.0009 (–0.0026, 0.0008), 0.3	–0. 0016 (–0.0027, –0.0004), 0.008
*PLAGL1/HYMAI*
Mean life childhood lead levels (birth to age 78months)	0.0016 (–0.0021, 0.0052), 0.4	0.0023 (–0.0046, 0.0091), 0.5	–0.0001 (–0.0045, 0.0044), 1.0
Prenatal lead levels (2nd or 3rd trimester)	–0.0023 (–0.0091, 0.0044), 0.5	–0. 0041 (–0.0160, 0.0078), 0.5	–0.0023 (–0.0104, 0.0058), 0.6
Neonatal lead levels (age 10 days)	0.0075 (0.0018, 0.0132), 0.01	0.0074 (–0.0001, 0.0150), 0.06	0.0030 (–0.0069, 0.0129), 0.6
Early childhood levels (age 3–30months)	0.0024 (–0.0011, 0.0059), 0.2	0.0055 (–0.0006, 0.0115), 0.08	0.00001 (–0.0042, 0.0042), 1.0
Middle childhood levels (age 33–78months)	0.0006 (–0.0028, 0.0040), 0.7	–0.0009 (–0.0071, 0.0053), 0.8	–0.0001 (–0.0043, 0.0042), 1.0
^***a***^Unstandardized regression coefficients. All models were adjusted for batch (first or second) and maternal cigarette smoking (none, one-half, 1 and 2 packs a day). Models of combined estimates for males and females are also adjusted for sex. ^***b***^Mean for each developmental period (early childhood) was derived by summing up lead levels for the participant and dividing by the number of observations.			

*Childhood lead levels and* PEG3 *DMR methylation*. Table 2 shows an association between mean lead concentrations across childhood and lower *PEG3* DMR methylation in adulthood (β = –0.0014; 95% CI: –0.0023, –0.0005, *p* = 0.002). These associations were primarily in males (β = –0.0024; 95% CI: –0.0038, –0.0009, *p* = 0.003) compared with females (β = –0.0009; 95% CI: –0.0020, 0.0003, *p* = 0.1). The cross-product term *p*-value for mean lead concentrations and sex was 0.09. This association in males corresponds to a 0.24% decrease in CpG methylation at the *PEG3* DMR, for every 1-μg/dL increase in lead concentration.

There were statistically significant inverse associations between *PEG3* DMR methylation and lead levels in early (β = –0.0012; 95% CI: –0.0020, –0.0004, *p* = 0.005) and middle childhood (β = –0.0013; 95% CI: –0.0021, –0.0005, *p* = 0.002). The associations were significant only in males during early (β = –0.0023; 95% CI: –0.0036, –0.0009, *p* = 0.002 for males and β = –0.0006; 95% CI: –0.0016, 0.0005, *p* = 0.3 for females) and middle childhood (β = –0.0018; 95% CI: –0.0031, –0.0004, *p* = 0.02 for males and β = –0.0009; 95% CI: –0.0020, 0.0001, *p* = 0.1 for females). Cross-product terms for early and middle childhood lead exposure and sex were *p* = 0.04 and 0.3, respectively. There were no statistically significant associations between prenatal or neonatal lead concentration and *PEG3* DMR methylation, although the direction of associations was largely similar to those of early and middle childhood.

Patterns of association observed in overall and sex-specific mean lead concentrations were also evident when maximum lead concentrations were considered (see Supplemental Material, Table S5). The association between the maximum lead concentration and decreased *PEG*3 DMR methylation in adulthood (β = –0.0007; 95% CI: –0.0012, –0.0003, *p* = 0.003) was also more apparent in males (β = –0.0013; 95% CI: –0.0021, –0.0006, *p*-value = 0.001) than in females (β = –0.0004; 95% CI: –0.0010, 0.0002, *p* = 0.3). Maximum lead concentrations in the prenatal and neonatal ages were too low for meaningful analyses.

To refine potential windows of vulnerability, nonstandardized regression coefficients were plotted for the associations between DMR methylation and averaged cumulative lead concentrations for each age at which lead was measured (Figure 3). Figure 3A confirms the association in Table 2 that higher lead exposure is associated with lower *PEG3* DMR methylation, and also shows that this association does not vary significantly after age 12–20 months to age 6.5 years, despite wide variation in lead concentrations during the observation period. These associations are male-specific.

*Childhood lead levels and* IGF2/H19 *DMR methylation.* Regression coefficients and *p-*values for the association between mean lead concentration and *IGF2/H19* DMR methylation in adulthood are also shown in Table 2. Mean childhood lead concentration was significantly associated with adult *IGF2/H19* DMR methylation (β = –0.0013; 95% CI: –0.0023, –0.0003, *p* = 0.01). This association may be stronger in females (β = –0.0017; 95% CI: –0.0029, –0.0006, *p* = 0.005) than in males (β = –0.0004; 95% CI: –0.0023, 0.0015, *p* = 0.7).

Associations for lead exposure and *IGF2/H19* DMR methylation were also found in early (β = –0.0016; 95% CI: –0.0027, –0.0005, *p* = 0.007) and middle childhood (β = –0.0016; 95% CI: –0.0027, –0.0004, *p* = 0.008) in females. These associations were weaker and less consistent in males, for early (β = 0.0004; 95% CI: –0.0014, 0.0022, *p* = 0.7) and middle childhood (β = –0.0009; 95% CI: –0.0026, 0.0008, *p* = 0.3). The *p*-values for cross-product terms for early and middle childhood lead levels and sex were 0.10 and 0.6, respectively. This age- and sex-specific pattern of association was also noted when maximum blood lead concentrations were evaluated (see Supplemental Material, Table S5). As with the *PEG3* DMR methylation, associations with prenatal and neonatal exposure were weaker although in the same direction.

Further exploration of cumulative lead concentrations suggests a female-specific association between lead exposure and lower methylation at the *IGF2/H19* DMR. The magnitude of the association between cumulative lead concentration and *IGF2/H19* DMR methylation was the same after 12–20 months (Figure 3B). As with *PEG3* DMR, this similarity persists for the entire observation period.

*Childhood lead levels and* PLAGL1/HYMAI *DMR methylation.* Unlike *IGF2/H19* and *PEG3*, which showed no evidence for association between neonatal lead exposure and DMR methylation, *PLAGL1/HYMAI* DMR methylation was positively associated with lead levels during this period (β = 0.0075; 95% CI: 0.0018, 0.0132, *p* = 0.01). The wide confidence intervals suggest that this association varied little between males (β = 0.0074; 95% CI: –0.0001, 0.0150, *p* = 0.06) and females (β = 0.0030; 95% CI: –0.0069, 0.0129, *p* = 0.6). No significant association was found between lead exposure and *PLAGL1/HYMAI* DMR methylation at any other age. Refined age-dependent and sex-specific analyses suggest that the magnitude of associations remained unaltered after ~ 12–20 months to the end of the observation period (Figure 3C).

## Discussion

The DMRs regulating monoallelic expression of imprinted genes are proposed to function as epigenetic archives of early exposure to environmental factors (Hoyo et al. 2009). Nevertheless, until now, no empirical data have demonstrated associations between early exposure to lead and adult CpG methylation at DMRs controlling the parent-of-origin silencing of imprinted genes. Environmentally induced DNA methylation changes at imprint DMRs are usually stable once established (Heijmans et al. 2008), and have been associated with common chronic diseases and conditions, including neurological disorders, obesity, type 2 diabetes, and some cancers (Azzi et al. 2013; Feinberg 2007; Hoyo et al. 2012; Ishida and Moore 2013).

We have undertaken an analysis of 22 DMRs regulating human imprinted genes, and evaluated relationships between DNA methylation in adulthood and lead exposure spanning from the first or early second trimester to age 6.5 years. Our key findings were that childhood lead exposure was associated with significantly lower DNA methylation levels at the DMR regulating *PEG3*. We also found modest but consistent associations between average lead concentration and decreased methylation of the *IGF2/H19* DMR, and higher DNA methylation levels at the *PLAGL1/HYMAI* DMR in relation to neonatal exposure. These data further indicated that although childhood lead exposure was associated with differences in *PEG3* DMR methylation in males and the *IGF2/H19* DMR methylation in females, the association between neonatal lead concentrations and *PLAGL1/HYMAI* DMR methylation may not be sex-specific. Notably, lead associations with DNA methylation of imprint regulatory elements at these three loci were found in lead measured before age 30 months, regardless of sex or DMR. These data support the contention that environmentally driven perturbations at these DMRs occur early. Furthermore, developmental differences between the sexes may dictate the patterns of gene regulation that ensue in response to early challenges with this heavy metal.

PEG3 *DMR methylation and early lead exposure.* Although childhood lead exposure has been associated with increased risk of neurodevelopmental disorders (Dietrich 2010), the mechanisms underlying these pathological conditions are poorly understood. *PEG3* plays a critical role in brain development, with expression mainly in the mesencephalon and pituitary gland; in the adult brain *PEG3* is found primarily in the hypothalamus and the pituitary gland (Li et al. 1999). In mouse models, *Peg3* also plays an important role in social and maternal nurturing behaviors, and paternal transmission of disrupted *Peg3* also leads to restricted growth (Chiavegatto et al. 2012; Li et al. 1999). In humans, hypermethylation at this locus has been associated with decreased gene expression of this tumor suppressor gene in cervical (Nye et al. 2013) and ovarian (Feng et al. 2008) cancers. In primary neuronal cell cultures derived from wild-type, p53-deficient, or Bax-deficient mice, overexpression of *Peg3* led to decreased neuronal viability via *p53* and *Bax* dependent pathways (Johnson et al. 2002). It is therefore possible that the male-specific reduced brain volume recently observed in these study participants (Cecil et al. 2008) may result, in part, from the dysregulation of *PEG3* during early development.

Interestingly, early-childhood but not prenatal or neonatal lead levels were associated with adulthood *PEG3* DMR hypomethylation, an association that may be specific to males. Because these DNA methylation marks are established early, it is possible that methylation differences observed were attributable to lead accumulated *in utero* and mobilized from soft tissue and bone after birth together with concurrent exposure. Alternatively, the reduced DNA methylation of the *PEG3* DMR marks may have been established postnatally (Loke et al. 2013). The latter possibility is consistent with human developmental studies suggesting that the first 1,000 days can dictate lifetime risk of common diseases (Victora et al. 2008). Discriminating between these possibilities requires larger studies with long-term follow-up.

IGF2/H19 *imprinted domain and early lead exposure.* The paternally expressed *insulin-like growth factor-2* (*IGF2*) is a commonly studied imprinted gene, and is frequently shown to be altered epigenetically by *in utero* environmental perturbations, and in cancer (Cruz-Correa et al. 2004, 2009; Cui et al. 2003; Heijmans et al. 2008; Hoyo et al. 2011, 2012; Murphy et al. 2012a). Dysregulation of the *IGF2/H19* domain was initially associated with Beckwith–Wiedemann syndrome (BWS) (Engel et al. 2000). Decreased DNA methylation at the *IGF2/H19* DMR has been associated with reduced *IGF2* expression in bladder cancer (Takai et al. 2001). This occurs when enhanced binding of the CTCF insulator protein to the normally unbound paternal allele (Nakagawa et al. 2001) blocks promoter interactions with downstream enhancers, thereby reducing gene expression (Hark et al. 2000; Kanduri et al. 2000). *Igf2* over-expression results in animal overgrowth (Sun et al. 1997), whereas gene repression results in restricted growth (DeChiara et al. 1990). IGF2 is also required for memory formation (Chen et al. 2011).

PLAGL1/HYMA1 *imprinted domain and early lead exposure.* A higher-order regulation of imprinted gene clusters is thought to exist and occur through epigenetic marks present at imprinting centers (Lewis and Reik 2006). Our finding that neonatal lead exposure is associated with increased methylation at the *PLAGL1/HYMAI (ZAC)* DMR regardless of sex is potentially of biological importance. In animals, microarray analysis shows that knockout of the mouse homolog, *Zac1 (Plagl1),* disrupts a network of coordinately regulated genes containing a large number that are also imprinted (Varrault et al. 2006). *In vitro* studies show induction of imprinted *Igf2*, *Cdkn1c, H19, Dlk1*, and *Mest* when *Zac1* is overexpressed (Varrault et al. 2006). Conversely, loss of *Zac1* expression in null mice results in inhibition of *Igf2, Cdkn1c, H19*, and *Dlk1* expression. Another imprinted gene network was identified by analyzing chromatin domains in other regions of the genome that interact with the *Igf2/H19* domain, *in vitro* (Zhao et al. 2006). The maternally expressed long noncoding *H19* RNA and the methyl-CpG–binding protein Mbd1 form a complex that regulates multiple imprinted genes by interacting with histone lysine methyltransferases. In mice, paternally expressed *Plagl1* is implicated in transient neonatal diabetes when overexpressed (Ma et al. 2004). In ovarian cell lines, *PLAGL1* was found to regulate *CDKN1C (p57^KIP2^)* expression and cell growth by inducing *LIT1* transcription in a methylation-dependent manner (Arima et al. 2005). Overexpression of *PLAGL1* induced *IGF2, H19*, and *CDKN1C* expression in a prostate cancer cell line (Ribarska et al. 2014). Together, these studies support a set of imprinted genes functioning in a network, coordinated in part by *Zac* (Finkielstain et al. 2009; Lui et al. 2008). Thus, environmentally induced epigenetic shifts of the *PLAGL1/HYMAI* regulatory DMR have the potential to alter network-wide imprinted gene expression. Studies with a larger number of DMRs are required to clarify the role of the *PLAGL1/HYMAI* DMR in the higher-order regulation of imprint clusters. Nevertheless, our findings are consistent with the idea that the far-reaching effects of early lead exposure may be mediated by stable, mitotically heritable epigenetic alterations in DMRs controlling imprinted gene expression.

A cautious interpretation of our findings is warranted. Although lead is known to target multiple organs, DNA methylation was measured using unfractionated peripheral blood collected in adulthood—the only accessible cell type—raising concerns about potential confounding by cell type, and other exposures during the life course that could not be evaluated. Another limitation of this study is the relatively small sample size, which reduced the precision of associations found. Assay limitations also precluded the measurement of DNA methylation for ~ 50% of CpGs within CpG-containing fragments. Because methylation values for CpG-containing fragments were averaged from individual CpG sites with similar methylation values and are *cis*-acting, such missing data should not alter our findings. The small amount of peripheral blood leukocyte DNA available for methylation analysis also precluded the determination of altered gene expression via other epigenetic mechanisms (e.g., histone modifications and chromatin structure changes); however, similar methylation changes at both the *IGF2/H19* and *PEG3* DMRs have been associated previously with altered gene expression in human cancers (Cui et al. 2003; Nye et al. 2013). Thus, our findings add preliminary support to accumulating evidence indicating that early lead exposure and gene-specific, epigenetic dysregulation of some imprinted gene DMRs may contribute to developmental abnormalities (Ishida and Moore 2013).

Our study also has major strengths. They include the determination of lead levels ~ 30 years before quantification of DNA methylation levels at imprinted gene DMRs. The numerous measurements of lead concentration during early development also facilitate estimating developmental windows in which lead exposure may exert its effects on regulatory DMRs. Furthermore, blood lead concentrations reflect both short- and longer-term exposure, including lead mobilized from physiological deposits.

To our knowledge, our findings represent the first attempt in humans to quantify associations between early lead exposure and DNA methylation alterations in adulthood at imprinted loci that are known experimentally to result in developmental and neurological disorders if perturbed early in development. Because lead exposure disproportionately affects those in the lower socioeconomic strata (Emerson 2012; Rai et al. 2012; Wright et al. 2008), our findings, if replicated in larger studies, may offer a potential explanation for observed DNA methylation differences among socioeconomic strata (Szyf 2012, 2013).

## Conclusions

Preventing lead exposure during vulnerable developmental windows remains sound policy. Nevertheless, effective therapeutic and public health strategies will depend on a better understanding of mechanisms underpinning the associations between lead exposure and the genesis of neurodevelopmental disorders and other poor health outcomes. Improved understanding should also guide policy regarding the highest tolerable limits in humans, a value currently unknown. Although the small sample size limits inference, this study provides preliminary evidence for significant associations between early lead exposure and DNA methylation at the regulatory regions of *PEG3, H19/IGF2*, and *PLAGL1/HYMAI*. Because these changes in the epigenome are acquired early, resultant shifts in the regulation of imprinted genes may contribute to increased risk of poor health outcomes (Ishida and Moore 2013; Murphy and Jirtle 2003). It remains unknown whether lead exposure previously associated with decreased gray matter volume (Cecil et al. 2008) and delinquent behavior (Dietrich et al. 2001) reported in this study population is mediated in part by the epigenetic alterations in imprinted gene regulatory elements, but this intriguing possibility needs to be investigated.

## Supplemental Material

(640 KB) PDF

## References

[r1] Arima T, Kamikihara T, Hayashida T, Kato K, Inoue T, Shirayoshi Y (2005). *ZAC, LIT1* (*KCNQ1OT1*) and *p57^KIP2^* (*CDKN1C*) are in an imprinted gene network that may play a role in Beckwith-Wiedemann syndrome.. Nucleic Acids Res.

[r2] Azzi S, Sas TC, Koudou Y, Le Bouc Y, Souberbielle JC, Dargent-Molina P (2013). Degree of methylation of *ZAC1* (*PLAGL1*) is associated with prenatal and post-natal growth in healthy infants of the EDEN mother child cohort.. Epigenetics.

[r3] Barlow DP (2011). Genomic imprinting: a mammalian epigenetic discovery model.. Annu Rev Genet.

[r4] Bellinger D, Leviton A, Needleman HL, Waternaux C, Rabinowitz M (1986). Low-level lead exposure and infant development in the first year.. Neurobehav Toxicol Teratol.

[r5] Bellinger D, Leviton A, Waternaux C, Needleman H, Rabinowitz M (1987). Longitudinal analyses of prenatal and postnatal lead exposure and early cognitive development.. N Engl J Med.

[r6] Bernal AJ, Dolinoy DC, Huang D, Skaar DA, Weinhouse C, Jirtle RL (2013). Adaptive radiation-induced epigenetic alterations mitigated by antioxidants.. FASEB J.

[r7] Binns HJ, Campbell C, Brown MJ, Centers for Disease Control and Prevention Advisory Committee on Childhood Lead Poisoning Prevention (2007). Interpreting and managing blood lead levels of less than 10 μg/dL in children and reducing childhood exposure to lead: recommendations of the Centers for Disease Control and Prevention Advisory Committee on Childhood Lead Poisoning Prevention.. Pediatrics.

[r8] Bolin CM, Basha R, Cox D, Zawia NH, Maloney B, Lahiri DK (2006). Exposure to lead and the developmental origin of oxidative DNA damage in the aging brain.. FASEB J.

[r9] Cecil KM Brubaker CJ Adler CM Dietrich KN Altaye M Egelhoff JC 2008 Decreased brain volume in adults with childhood lead exposure. PLoS Med 5 e112; doi:10.1371/journal.pmed.0050112 18507499 PMC2689675

[r10] Chen DY, Stern SA, Garcia-Osta A, Saunier-Rebori B, Pollonini G, Bambah-Mukku D (2011). A critical role for IGF-II in memory consolidation and enhancement.. Nature.

[r11] Chiavegatto S, Sauce B, Ambar G, Cheverud JM, Peripato AC (2012). Hypothalamic expression of *Peg3* gene is associated with maternal care differences between SM/J and LG/J mouse strains.. Brain Behav.

[r12] Cruz-Correa M, Cui H, Giardiello FM, Powe NR, Hylind L, Robinson A (2004). Loss of imprinting of insulin growth factor II gene: a potential heritable biomarker for colon neoplasia predisposition.. Gastroenterology.

[r13] Cruz-Correa M, Zhao R, Oviedo M, Bernabe RD, Lacourt M, Cardona A (2009). Temporal stability and age-related prevalence of loss of imprinting of the insulin-like growth factor-2 gene.. Epigenetics.

[r14] Cui H, Cruz-Correa M, Giardiello FM, Hutcheon DF, Kafonek DR, Brandenburg S (2003). Loss of *IGF2* imprinting: a potential marker of colorectal cancer risk.. Science.

[r15] DeChiara TM, Efstratiadis A, Robertson EJ (1990). A growth-deficiency phenotype in heterozygous mice carrying an insulin-like growth factor II gene disrupted by targeting.. Nature.

[r16] Dietrich KN (2010). Environmental toxicants.. In: Pediatric Neuropsychology: Research, Theory, and Practice (Yeates KO, Ris MD, Taylor HG, Pennington BF, eds). 2nd ed.

[r17] Dietrich KN, Berger OG, Succop PA (1993). Lead exposure and the motor developmental status of urban six-year-old children in the Cincinnati Prospective Study.. Pediatrics.

[r18] Dietrich KN, Krafft KM, Bornschein RL, Hammond PB, Berger O, Succop PA (1987). Low-level fetal lead exposure effect on neurobehavioral development in early infancy.. Pediatrics.

[r19] Dietrich KN, Ris MD, Succop PA, Berger OG, Bornschein RL (2001). Early exposure to lead and juvenile delinquency.. Neurotoxicol Teratol.

[r20] Dolinoy DC, Huang D, Jirtle RL (2007). Maternal nutrient supplementation counteracts bisphenol A-induced DNA hypomethylation in early development.. Proc Natl Acad Sci USA.

[r21] Dolinoy DC Weidman JR Waterland RA Jirtle RL 2006 Maternal genistein alters coat color and protects *A^vy^* mouse offspring from obesity by modifying the fetal epigenome. Environ Health Perspect 114 567 572; doi:10.1289/ehp.8700 16581547 PMC1440782

[r22] Edwards CA Rens W Clarke O Mungall AJ Hore T Graves JA 2007 The evolution of imprinting: chromosomal mapping of orthologues of mammalian imprinted domains in monotreme and marsupial mammals. BMC Evol Biol 7 157; doi:10.1186/1471-2148-7-157 17822525 PMC2042987

[r23] Emerson E (2012). Deprivation, ethnicity and the prevalence of intellectual and developmental disabilities.. J Epidemiol Community Health.

[r24] Engel JR, Smallwood A, Harper A, Higgins MJ, Oshimura M, Reik W (2000). Epigenotype-phenotype correlations in Beckwith-Wiedemann syndrome.. J Med Genet.

[r25] Faulk C, Barks A, Liu K, Goodrich JM, Dolinoy DC (2013). Early-life lead exposure results in dose- and sex-specific effects on weight and epigenetic gene regulation in weanling mice.. Epigenomics.

[r26] Feinberg AP (2007). Phenotypic plasticity and the epigenetics of human disease.. Nature.

[r27] Feng W, Marquez RT, Lu Z, Liu J, Lu KH, Issa JP (2008). Imprinted tumor suppressor genes *ARHI* and *PEG3* are the most frequently down-regulated in human ovarian cancers by loss of heterozygosity and promoter methylation.. Cancer.

[r28] Finkelstein Y, Markowitz ME, Rosen JF (1998). Low-level lead-induced neurotoxicity in children: an update on central nervous system effects.. Brain Res Brain Res Rev.

[r29] Finkielstain GP, Forcinito P, Lui JC, Barnes KM, Marino R, Makaroun S (2009). An extensive genetic program occurring during postnatal growth in multiple tissues.. Endocrinology.

[r30] Hark AT, Schoenherr CJ, Katz DJ, Ingram RS, Levorse JM, Tilghman SM (2000). CTCF mediates methylation-sensitive enhancer-blocking activity at the *H19/Igf2* locus.. Nature.

[r31] Heijmans BT, Tobi EW, Stein AD, Putter H, Blauw GJ, Susser ES (2008). Persistent epigenetic differences associated with prenatal exposure to famine in humans.. Proc Natl Acad Sci USA.

[r32] Hoyo C, Fortner K, Murtha AP, Schildkraut JM, Soubry A, Demark-Wahnefried W (2012). Association of cord blood methylation fractions at imprinted insulin-like growth factor 2 (*IGF2*), plasma IGF2, and birth weight.. Cancer Causes Control.

[r33] Hoyo C, Murphy SK, Jirtle RL (2009). Imprint regulatory elements as epigenetic biosensors of exposure in epidemiological studies.. J Epidemiol Community Health.

[r34] Hoyo C, Murtha AP, Schildkraut JM, Jirtle RL, Demark-Wahnefried W, Forman MR (2011). Methylation variation at *IGF2* differentially methylated regions and maternal folic acid use before and during pregnancy.. Epigenetics.

[r35] Ishida M, Moore GE (2013). The role of imprinted genes in humans.. Mol Aspects Med.

[r36] Johnson MD, Wu X, Aithmitti N, Morrison RS (2002). Peg3/Pw1 is a mediator between p53 and Bax in DNA damage-induced neuronal death.. J Biol Chem.

[r37] Kanduri C, Pant V, Loukinov D, Pugacheva E, Qi CF, Wolffe A (2000). Functional association of CTCF with the insulator upstream of the *H19* gene is parent of origin-specific and methylation-sensitive.. Curr Biol.

[r38] Lewis A, Reik W (2006). How imprinting centres work.. Cytogenet Genome Res.

[r39] Li L, Keverne EB, Aparicio SA, Ishino F, Barton SC, Surani MA (1999). Regulation of maternal behavior and offspring growth by paternally expressed *Peg3*.. Science.

[r40] Loke YJ, Galati JC, Morley R, Joo EJ, Novakovic B, Li X (2013). Association of maternal and nutrient supply line factors with DNA methylation at the imprinted *IGF2/H19* locus in multiple tissues of newborn twins.. Epigenetics.

[r41] Lui JC, Finkielstain GP, Barnes KM, Baron J (2008). An imprinted gene network that controls mammalian somatic growth is down-regulated during postnatal growth deceleration in multiple organs.. Am J Physiol Regul Integr Comp Physiol.

[r42] Luo M, Xu Y, Cai R, Tang Y, Ge MM, Liu ZH (2014). Epigenetic histone modification regulates developmental lead exposure induced hyperactivity in rats.. Toxicol Lett.

[r43] Ma D, Shield JP, Dean W, Leclerc I, Knauf C, Burcelin RR (2004). Impaired glucose homeostasis in transgenic mice expressing the human transient neonatal diabetes mellitus locus, TNDM.. J Clin Invest.

[r44] Murphy SK, Adigun A, Huang Z, Overcash F, Wang F, Jirtle RL (2012a). Gender-specific methylation differences in relation to prenatal exposure to cigarette smoke.. Gene.

[r45] Murphy SK Huang Z Hoyo C 2012b Differentially methylated regions of imprinted genes in prenatal, perinatal and postnatal human tissues. PLoS One 7 e40924; doi:10.1371/journal.pone.0040924 22808284 PMC3396645

[r46] Murphy SK, Jirtle RL (2003). Imprinting evolution and the price of silence.. Bioessays.

[r47] Nakagawa H, Chadwick RB, Peltomaki P, Plass C, Nakamura Y, de La Chapelle A (2001). Loss of imprinting of the insulin-like growth factor II gene occurs by biallelic methylation in a core region of *H19*-associated CTCF-binding sites in colorectal cancer.. Proc Natl Acad Sci USA.

[r48] Nye MD Hoyo C Huang Z Vidal AC Wang F Overcash F 2013 Associations between methylation of *paternally expressed gene 3* (*PEG3*), cervical intraepithelial neoplasia and invasive cervical cancer. PLoS One 8 e56325; doi:10.1371/journal.pone.0056325 23418553 PMC3571954

[r49] Pilsner JR Hu H Ettinger A Sánchez BN Wright RO Cantonwine D 2009 Influence of prenatal lead exposure on genomic methylation of cord blood DNA. Environ Health Perspect 117 1466 1471; doi:10.1289/ehp.0800497 19750115 PMC2737027

[r50] Rai D Lewis G Lundberg M Araya R Svensson A Dalman C 2012 Parental socioeconomic status and risk of offspring autism spectrum disorders in a Swedish population-based study. J Am Acad Child Adolesc Psychiatry 51 467 476.e6; doi:10.1016/j.jaac.2012.02.012 22525953

[r51] R Core Team (2013). R: A Language and Environment for Statistical Computing.. http://www.R-project.org/.

[r52] Reichard JF, Schnekenburger M, Puga A (2007). Long term low-dose arsenic exposure induces loss of DNA methylation.. Biochem Biophys Res Commun.

[r53] Reik W, Walter J (2001). Genomic imprinting: parental influence on the genome.. Nat Rev Genet.

[r54] Ribarska T, Goering W, Droop J, Bastian KM, Ingenwerth M, Schulz WA (2014). Deregulation of an imprinted gene network in prostate cancer.. Epigenetics.

[r55] Roda SM, Greenland RD, Bornschein RL, Hammond PB (1988). Anodic stripping voltammetry procedure modified for improved accuracy of blood lead analysis.. Clin Chem.

[r56] Sanders T, Liu Y, Buchner V, Tchounwou PB (2009). Neurotoxic effects and biomarkers of lead exposure: a review.. Rev Environ Health.

[r57] Skaar DA, Li Y, Bernal AJ, Hoyo C, Murphy SK, Jirtle RL (2012). The human imprintome: regulatory mechanisms, methods of ascertainment, and roles in disease susceptibility.. ILAR J.

[r58] Sun FL, Dean WL, Kelsey G, Allen ND, Reik W (1997). Transactivation of *Igf2* in a mouse model of Beckwith–Wiedemann syndrome.. Nature.

[r59] Szyf M (2012). The early-life social environment and DNA methylation.. Clin Genet.

[r60] Szyf M (2013). The genome- and system-wide response of DNA methylation to early life adversity and its implication on mental health.. Can J Psychiatry.

[r61] Takai D, Gonzales FA, Tsai YC, Thayer MJ, Jones PA (2001). Large scale mapping of methylcytosines in CTCF-binding sites in the human *H19* promoter and aberrant hypomethylation in human bladder cancer.. Hum Mol Genet.

[r62] Takiguchi M, Achanzar WE, Qu W, Li G, Waalkes MP (2003). Effects of cadmium on DNA-(Cytosine-5) methyltransferase activity and DNA methylation status during cadmium-induced cellular transformation.. Exp Cell Res.

[r63] Varrault A, Gueydan C, Delalbre A, Bellmann A, Houssami S, Aknin C (2006). Zac1 regulates an imprinted gene network critically involved in the control of embryonic growth.. Dev Cell.

[r64] Victora CG, Adair L, Fall C, Hallal PC, Martorell R, Richter L (2008). Maternal and child undernutrition: consequences for adult health and human capital.. Lancet.

[r65] Waterland RA, Jirtle RL (2003). Transposable elements: targets for early nutritional effects on epigenetic gene regulation.. Mol Cell Biol.

[r66] Waterland RA Kellermayer R Laritsky E Rayco-Solon P Harris RA Travisano M 2010 Season of conception in rural Gambia affects DNA methylation at putative human metastable epialleles. PLoS Genet 6 e1001252; doi:10.1371/journal.pgen.1001252 21203497 PMC3009670

[r67] Wilson AS, Power BE, Molloy PL (2007). DNA hypomethylation and human diseases.. Biochim Biophys Acta.

[r68] Winneke G, Krämer U, Brockhaus A, Ewers U, Kujanek G, Lechner H (1983). Neuropsychological studies in children with elevated tooth-lead concentrations. II. Extended study.. Int Arch Occup Environ Health.

[r69] Woodfine K Huddleston JE Murrell A 2011 Quantitative analysis of DNA methylation at all human imprinted regions reveals preservation of epigenetic stability in adult somatic tissue. Epigenetics Chromatin 4 1; doi:10.1186/1756-8935-4-1 21281512 PMC3038880

[r70] Wright JP Dietrich KN Ris MD Hornung RW Wessel SD Lanphear BP 2008 Association of prenatal and childhood blood lead concentrations with criminal arrests in early adulthood. PLoS Med 5 e101; doi:10.1371/journal.pmed.0050101 18507497 PMC2689664

[r71] Wright RO Schwartz J Wright RJ Bollati V Tarantini L Park SK 2010 Biomarkers of lead exposure and DNA methylation within retrotransposons. Environ Health Perspect 118 790 795; doi:10.1289/ehp.0901429 20064768 PMC2898855

[r72] Zahran S, Mielke HW, Weiler S, Berry KJ, Gonzales C (2009). Children’s blood lead and standardized test performance response as indicators of neurotoxicity in metropolitan New Orleans elementary schools.. Neurotoxicology.

[r73] Zhao Z, Tavoosidana G, Sjölinder M, Göndör A, Mariano P, Wang S (2006). Circular chromosome conformation capture (4C) uncovers extensive networks of epigenetically regulated intra- and interchromosomal interactions.. Nat Genet.

