# Supplementary material for: Lead Exposure during Early Human Development and DNA Methylation of Imprinted Gene Regulatory Elements in Adulthood
Source: Environ Health Perspect. 2015 Jun 26;124(5):666–73. doi: 10.1289/ehp.1408577 (PMC4858407; doi:10.1289/ehp.1408577)
Supplement: (640 KB) PDF [file ehp.1408577.s001.acco.pdf]

Environ Health Perspect
DOI: 10.1289/ehp.1408577

Note to Readers: *EHP* strives to ensure that all journal content is accessible to all readers. However, some figures and Supplemental Material published in *EHP* articles may not conform to 508 standards due to the complexity of the information being presented. If you need assistance accessing journal content, please contact ehp508@niehs.nih.gov. Our staff will work with you to assess and meet your accessibility needs within 3 working days.

Supplemental Material

Lead Exposure during Early Human Development and DNA Methylation of Imprinted Gene Regulatory Elements in Adulthood

Yue Li, Changchun Xie, Susan K. Murphy, David Skaar, Monica Nye, Adriana C. Vidal, Kim M. Cecil, Kim N. Dietrich, Alvaro Puga, Randy L. Jirtle, and Cathrine Hoyo

Table of Contents

Table S1. Amplicon data of DMRs with methylation analyzed by MassArray

Table S2. PCR conditions for DNA methylation analysis for 22 DMRs

Table S3. Imprinted gene DMR methylation analysis for the 105 participants with varying levels of lead exposure

Table S4. Correlation coefficients and p-values for the relationship among lead levels from pre- and post-natal period, age 1-78 months

Table S5. Adjusted regression coefficients for the association between maximum lead exposure and *PEG3, IGF2/H19*, and *PLAGL1 /HYMAI* DMR methylation

Table S1. Amplicon data of DMRs with methylation analyzed by MassArray

**Gene name**	**CpG site, CpG position, and Comments**																							
DIRAS3	CpG Sites	1,2	3	4	5	6	7	8	9	10	11	12												
	CpG positions	37,39	45	58	68	109	143	161	200	266	282	320												
	Comments		Ex - LM	Ex - SN			Ex - LM	Ex - SN		Ex - FR	Ex - SN													
IL1-alpha	CpG Sites	1	2	3	4	5,6	7	8	9,10	11	12													
	CpG positions	42	98	124	132	146,149	157	211	351,353	391	400													
	Comments	D7 D10	Ex - FR	Ex - FR	Ex - FR		Ex - FR	D1 D10	Ex - FR		D1 D7													
NAP1L5	CpG Sites	1	2,3	4,5	6,7,8	9	10,11,12	13	14	15	16	17	18	19	20	21	22	23,24	25,26	27	28	29,30	31,32	33,34
	CpG positions	38	103,107	139,141	154,160, 162	169	178,180, 184	188	194	204	210	233	250	272	293	298	316	331,334	340,343	352	370	377,382	404,406	425,427
	Comments	Ex - SN	Ex - SN			Ex - SM		Ex - SM	Ex - SN		Ex - SN				Ex - SN	Ex - SN	Ex - SN			Ex - FR	Ex - SN			
FAM50B	CpG Sites	1,2	3	4	5,6,7	8,9	10,11	12, 13,14,15	16	17,18	19	20,21,22												
	CpG positions	39,47	57	62	83,90,92	100,105	121,134	150,152, 160,164	192	237,242	263	268,276, 280												
	Comments						Ex - HM			Ex - FR														
PLALG1/HYMAI	CpG Sites	1	2	3	4	5	6	7	8															
	CpG positions	64	145	171	188	205	223	400	413															
	Comments			Ex – FR	Ex - SN	Ex - ND	Ex - FR	Ex - SN	Ex - HM															
GRB10	CpG Sites	1	2	3	4	5	6	7	8,9,10,11	12,13	14,15	16	17	18	19,20	21								
	CpG positions	66	104	117	193	223	247	255	272,274, 278,280	290,300	306,309	314	335	341	360,362	379								
	Comments	Ex - FR			Ex - LM			Ex - SN			Ex - SN	Ex - SN		Ex - FR										
PEG10	CpG Sites	1	2,3	4	5	6	7	8	9	10	11													
	CpG positions	39	53,55	68	77	97	108	117	126	159	173													
	Comments							Ex - FR		Ex - LM														
SGCE	CpG Sites	1	2,3	4	5	6	7	8	9	10	11	12	13	14	15	16								
	CpG positions	36	52,55	87	102	135	176	206	251	266	296	311	341	386	401	431								
	Comments	Ex - FR	Ex - OL4	Ex - FR	Ex - OL2	Ex - SN			Ex - SN	Ex - SN	Ex - SN	Ex - SN	Ex - SN	Ex - SN	Ex - SN	Ex - SN								
MESTIT1/MEST	CpG Sites	1	2	3	4,5	6	7,8,9,10, 11	12	13,14	15, 16, 17,18	19	20	21,22	23	24,25	26	27							
	CpG positions	78	103	129	155,164	168	176,181, 185,189, 196	214	231,237	241,246, 255,257	278	287	298,300	314	320,326	333	352							
	Comments	Ex - SN	Ex - LM	Ex - SN	Ex - OL7	Ex - FR	Ex - HM	Ex – OL4		Ex - HM				Ex - SN		Ex - LM	Ex - SN							
IGF2/H19	CpG Sites	1	2	3	4	5																		
	CpG positions	42	50	207	221	254																		
	Comments	Ex - LM																						
IGF2	CpG Sites	1,2	3	4	5	6	7	8	9	10														
	CpG positions	43,46	62	161	181	190	271	289	386	423														
	Comments	Ex - FR	Ex - FR	Ex - FR	Ex - FR	Ex - FR	Ex - FR	Ex - FR	Ex - FR	Ex – FR														
KvDMR	CpG Sites	1	2	3,4,5	6	7	8,9	10,11,12	13,14	15	16	17,18	19	20	21	22	23	24	25	26,27				
	CpG positions	47	57	60,62,65	69	87	93,98	108,111, 117	120,125	129	151	171,174	186	201	216	233	244	259	278	287,290				
	Comments		Ex - FR	Ex - SN		Ex - SN			Ex - SN				Ex - LM			Ex - SN	Ex - FR			Ex - SN				

Table S1 Cont. Amplicon data of DMRs with methylation analyzed by MassArray

RB1	CpG Sites	1	2	3	4	5	6,7	8	9,10	11	12	13												
**CpG position**	CpG positions	46	73	106	129	146	155,158	167	179,182	190	200	217												
	Comments		D4		D2	Ex - LM		Ex - LM		Ex – FR														
DLK1/MEG3	CpG Sites	1	2	3	4	5	6	7,8	9,10	11	12	13	14	15,16	17	18	19,20							
**CpG position**	CpG positions	50	75	94	101	123	141	154,157	169,172	189	225	269	297	323,326	349	357	377,380							
	Comments	Ex - HM	Ex - FR	Ex - FR		Ex - FR	Ex - LM	Ex - FR	Ex - FR	Ex – FR	Ex - LM	Ex - FR		Ex - FR	Ex - FR	Ex - LM	Ex - HM							
MEG3	CpG Sites	1,2	3	4	5	6	7	8,9,10	11	12,13	14	15	16	17	18	19	20,21	22	23	24,25	26,27,28	29,30,31		
**CpG position**	CpG positions	37,41	66	81	93	114	120	127,130, 147	160	182,187	203	211	249	269	282	310	315,318	336	344	379,392	401,410, 414	421,426, 429		
	Comments			Ex - LM	D14	D17	Ex - FR	Ex - HM	Ex - LM		Ex - SN			Ex - LM	D4	Ex - FR		D5		Ex - HM				
MAGEL2	CpG Sites	1,2	3,4	5	6	7	8	9	10	11	12,13	14												
**CpG position**	CpG positions	41,47	62,71	100	107	122	134	143	152	182	191,196	247												
	Comments	D2	D1		Ex - SN	D8			D5	Ex - SN														
NDN	CpG Sites	1	2	3	4	5	6	7,8,9	10,11	12,13	14	15	16,17	18	19,20	21	22,23	24	25	26,27	28			
**CpG position**	CpG positions	54	66	76	96	107	154	164,166,1 69	181,184	193,200	215	241	247,251	274	302,304	313	321,325	331	350	359,361	434			
	Comments	Ex - FR	Ex - SN	Ex - FR	Ex - FR	Ex - SN	Ex - SN						Ex - SN			Ex - SN	Ex - SN	Ex - LM	Ex - HM					
SNRPN	CpG Sites	1	2	3	4	5,6	7	8	9	10	11													
**CpG position**	CpG positions	178	192	346	352	368,371	377	383	404	422	464													
	Comments	Ex - SN			Ex - SN	Ex - SN	Ex - SN	Ex - SN	Ex - SN		Ex - HM													
PEG3/ZIM2	CpG Sites	1	2	3	4	5,6	7	8	9	10	11	12												
**CpG position**	CpG positions	50	58	77	89	132,136	144	178	215	254	274	282												
	Comments	Ex - SN			Ex - SN		Ex - SN	Ex - SN		Ex - LM	D6	Ex - SN												
BLCAP	CpG Sites	1	2	3,4	5,6	7	8,9,10,11	12,13	14	15,16	17	18	19	20	21,22	23	24	25	26	27				
**CpG position**	CpG positions	41	58	65,74	81,91	99	105,107,1 14,116	129,133	146	152,154	192	203	245	252	292,295	337	359	390	435	476				
	Comments					Ex - SN	Ex - FR		Ex - FR		Ex - HM		Ex- LM			Ex - LM	Ex- SN	Ex - FR		Ex - LM				
L3MBTL	CpG Sites	1	2	3,4	5,6	7,8,9	10	11,12,13	14,15	16	17	18	19	20										
**CpG position**	CpG positions	37	62	75,81	99,107	117,120,1 25	151	174,176,1 82	195,202	234	241	254	269	287										
	Comments		Ex - SN			Ex - SN					Ex – SN			Ex - LM										
GNAS	CpG Sites	1	2	3	4,5,6	7,8	9,10,11	12,13	14,15	16	17	18	19											
**CpG position**	CpG positions	47	71	79	105,108, 111	135,138	143,145,1 51	186,189	205,208	253	276	290	331											
	Comments	Ex- SN	Ex - SN	Ex - SN		Ex - SN		Ex - SN		Ex - SN	Ex - SN	Ex - SN	Ex - FR											

Fragments containing CpG sites are given, listing the sites contained in each fragment, and positions of CpGs within amplicons. Fragments with duplicate masses are indicated by ‘D’. Fragments that cannot be analyzed due to overlap of non-methylated or methylated forms with non-CpG fragments and low or high mass outside the detection range are indicated ‘SN’, ‘SM’, ‘LM’, and ‘HM’, respectively. Fragments excluded from analysis by failure rate >5% are indicated ‘FR’.

Table S2. PCR conditions for DNA methylation analysis for 22 DMRs

Temperature	Frequency	Activity
95°C 5 min	X 1	Polymerase activation
94°C 25 s<br>66°C 25 s<br>72°C 80 s	X 1	Touchdown cycle 1
94°C 25 s<br>64°C 25 s<br>72°C 80 s	X 1	Touchdown cycle 2
94°C 25 s<br>62°C 25 s<br>72°C 80 s	X 1	Touchdown cycle 3
94°C 25 s<br>60°C 25 s<br>72°C 60 s	X 39	Amplification
72°C 10 min	X 1	Final extension

Table S3. Imprinted gene DMR methylation analysis for 105 participants with varying levels of lead exposure

GENE	PRIMER SEQUENCES	CHR	[a] START	[a] END	[b] I/A fragments	[c] Methylation Mean (%, sd)
DIRAS3	aggaagagagTGAAATTGGAATTAGGTTTTTTTTG	1	68515792	68516128	5/6	50.8 (15.4)
	cagtaatacgactcactatagggagaaggctACCAACCTAACTCATAAACATCACC					
I1L-alpha	aggaagagagGATGTGAGGATAATATTTTTGTTGAT	2	113542004	113542503	5/10	46.3 (7.2)
	cagtaatacgactcactatagggagaaggctACCAATTCACCCTAAAACACAAT					
NAP1L5	aggaagagagTTGGGAATAGGGTGTTTGTTTAATA	4	89618738	89619182	12/12	36.7 (4.5)
	cagtaatacgactcactatagggagaaggctCTAAAACTCCTCAACCATCTAACCA					
FAM50B	aggaagagagAGAAGAGAGTTAGGAGTTGGTATTTTTTTTAGGG	6	3849549	3849893	9/10	45.2 (4.1)
	cagtaatacgactcactatagggagaaggctCAGTAATACGACTCACTATAGGGAGAAGG CTAAATAACCACCAACCTAACAATATC					
PLAGL1/HYMAI	aggaagagagGAAAAAGTTTGTTTTAAGTAATAATGGGAT	6	144328445	144328885	2/4	58.6 (10.9)
	cagtaatacgactcactatagggagaaggctAAAAAACCAAAACCTCAATAAAACC					
GRB10	aggaagagagTGAAGTGATTAGTATATAGTAGATGTTTGG	7	50850662	50851107	9/10	46.5 (5.1)
	cagtaatacgartcactatagggagaaggctAATCCCTAATTCTCATAACAACCCT					
PEG10	aggaagagagAGGTGTGGGATTTTATTTTTTTTGT	7	9428S845	94286061	8/9	49.2 (4.9)
	cagtaatacgactcactatagggagaaggctCAAACCTTTAAAACTTAATTTCCCC					
SGCE	aggaagagagAGTTAGGGTTTAGGTAAGGAAGGTG	7	94287476	94287939	3/4	40.4 (3.6)
	cagtaatacgactcactatagggagaaggctCCCCTCTAAAATAAACTACCCAAAC					
MESTIT1/MEST	aggaagagagGGGTTTAGAGGTATAAGAAAGAGGG	7	130130648	130131063	5/6	49.3 (5.9)
	cagtaatacgactcactatagggagaaggctTTTCTAAAAACAACCAAACCCCTAC					
IGF2/H19	aggaagagagTATTTTGAGGTTGGGGGATATTA	11	2130112	2130388	4/4	74.1 (3.0)
	cagtaatacgactcactatagggagaaggctCTCCCTCAACAAAAACTAACAAATC					
IGF2	aggaagagagGAGGGGGTTTATTTTTTTAGGAAGT	11	2169100	2169551	9/10	41.2 (4.8)
	cagtaatacgactcactatagggagaaggctATACACCCCAAACCTAAACCCT					
KvDMR	aggaagagagTTTGGTAGGATTTTGTTGAGGAGTTTT	11	2721161	2721464	11/11	46.4 (2.7)
	cagtaatacgactcactatagggagaaggctCTCACACCCAACCAATACCTCATAC					
RB1	aggaagagagGTTTTGGGGTTATTGGTTTGGT	13	48892886	48893124	8/9	47.5 (5.3)
	cagtaatacgactcactatagggagaaggctAAACAACAACAAATCCCTTTCTACA					
DLK1/MEG3	aggaagagagAGTTTTATAGGTTGTAAAGGGGGTG	14	101290922	101291330	2/9	59.8 (8.0)
	cagtaatacgactcactatagggagaaggctATAAACAAAAAACCCCAAAAAAAAC					
MEG3	aggaagagagTTGTGATAAGGTTAGTGAGGGGTTA	14	101293947	101294390	9/11	73.8 (7.3)
	cagtaatacgactcactatagggagaaggctCCAACCAAAACCCACCTATAACTAC					
MAGEL2	aggaagagagGGGAGTTGAAGGATGTATTATTAGGA	15	23892485	23892757	5/5	39.9 (7.7)
	cagtaatacgactcactatagggagaaggctAATCCACCTCCAATTAACTTACAAA					
NDN	aggaagagagGGGTTGTATTTGAGGTTAATTAGTTTG	15	23931458	23931911	9/9	61.7 (6.2)
	cagtaatacgactcactatagggagaaggctCTCTAACCTCCTCCAAAACrTCTCT					
SNRPN	aggaagagagTTTATTTATTGTGGTGTTTTAAAAGGAA	15	25068397	25068876	3/3	59.0 (7.7)
	cagtaatacgactcactatagggagaaggctAACAAAAAACAACTAACCTTACCCC					
PEG3,/ZIM2	aggaagagagTATTGGGTGTTATTTTTTATGAGGG	19	57350715	57351051	5/5	43.3 (3..2)
	cagtaatacgactcactatagggagaaggctTCTACTACCAACCAACCAAAACAAC					
BLCAP	aggaagagagTTATGGTTTTAAGAATGGTAGGTGG	20	36149241	36149737	10/13	64.9 (7.8)
	cagtaatacgactcactatagggagaaggctTCTATAAAACAAATAACCCCTCCCT					
L3MBTL	aggaagagagGTGTTTTGGATGTGTTTTGTTTTTT	20	42142508	42142820	9/9	38.3 (3.8)
	cagtaatacgartcartatagggagaaggctTAAAACCCATCATCTAAACCAACTT					
GNAS	aggaagagagGTTTTAGAGTTTTAGGGAAGGGGAG	20	57415713	57416072	3/4	43.0 (5.8)
	cagtaatacgactcactatagggagaaggctATCCCAAACTAACCAACTAAACCTC					

[a] Feb 2009, GRC37/hg19 build
[b] Included/Analyzable cleavage fragments per DMR, based on 5% failure cutoff limit.
[c] DMR methylation for all lead exposed participants.

Table S4. Correlation coefficients and p-values for the relationship among lead levels from pre- and post-natal period, age 1-78 months

**Age Pb measured (months)**	**Pre-natal**	**1**	**3**	**6**	**9**	**12**	**15**	**18**	**21**	**24**	**27**	**30**	**33**	**36**	**39**	**42**	**45**	**48**	**51**	**54**	**57**	**60**	**66**	**72**	**78**
**Prenatal**	1																								
**1**	0.19<br>0.06	1																							
**3**	0.24<br>0.02	0.61<br><.0001	1																						
**6**	0.30<br>0.00	0.33<br>0.00	0.46<br><.0001	1																					
**9**	0.21<br>0.04	0.31<br>0.00	0.37<br><.0001	0.71<br><.0001	1																				
**12**	0.27<br>0.01	0.25<br>0.01	0.43<br><.0001	0.46<br><.0001	0.64<br><.0001	1																			
**15**	0.17<br>0.09	0.27<br>0.01	0.36<br>0.00	0.45<br><.0001	0.58<br><.0001	0.67<br><.0001	1																		
**19**	0.28<br>0.01	0.19<br>0.04	0.28<br>0.00	0.59<br><.0001	0.69<br><.0001	0.66<br><.0001	0.74<br><.0001	1																	
**21**	0.29<br>0.00	0.26<br>0.01	0.17<br>0.08	0.40<br><.0001	0.56<br><.0001	0.58<br><.0001	0.58<br><.0001	0.74<br><.0001	1																
**24**	0.24<br>0.02	0.26<br>0.01	0.21<br>0.03	0.37<br><.0001	0.62<br><.0001	0.67<br><.0001	0.73<br><.0001	0.76<br><.0001	0.81<br><.0001	1															
**27**	0.28<br>0.01	0.25<br>0.01	0.28<br>0.00	0.44<br><.0001	0.60<br><.0001	0.67<br><.0001	0.74<br><.0001	0.75<br><.0001	0.71<br><.0001	0.86<br><.0001	1														
**30**	0.19<br>0.07	0.17<br>0.07	0.22<br>0.02	0.42<br><.0001	0.53<br><.0001	0.53<br><.0001	0.62<br><.0001	0.71<br><.0001	0.65<br><.0001	0.77<br><.0001	0.83<br><.0001	1													
**33**	0.25<br>0.01	0.24<br>0.01	0.26<br>0.01	0.43<br><.0001	0.52<br><.0001	0.53<br><.0001	0.55<br><.0001	0.65<br><.0001	0.67<br><.0001	0.71<br><.0001	0.76<br><.0001	0.84<br><.0001	1												
**36**	0.25<br>0.02	0.26<br>0.01	0.33<br>0.00	0.38<br><.0001	0.49<br><.0001	0.53<br><.0001	0.58<br><.0001	0.58<br><.0001	0.60<br><.0001	0.71<br><.0001	0.78<br><.0001	0.80<br><.0001	0.89<br><.0001	1											
**39**	0.22<br>0.03	0.22<br>0.02	0.22<br>0.02	0.33<br>0.00	0.42<br><.0001	0.53<br><.0001	0.59<br><.0001	0.61<br><.0001	0.60<br><.0001	0.72<br><.0001	0.78<br><.0001	0.77<br><.0001	0.82<br><.0001	0.91<br><.0001	1										
**42**	0.29<br>0.00	0.21<br>0.03	0.23<br>0.02	0.47<br><.0001	0.52<br><.0001	0.54<br><.0001	0.57<br><.0001	0.68<br><.0001	0.63<br><.0001	0.70<br><.0001	0.75<br><.0001	0.79<br><.0001	0.82<br><.0001	0.86<br><.0001	0.90<br><.0001	1									
**45**	0.32<br>0.00	0.26<br>0.01	0.28<br>0.00	0.43<br><.0001	0.46<br><.0001	0.54<br><.0001	0.55<br><.0001	0.65<br><.0001	0.64<br><.0001	0.65<br><.0001	0.73<br><.0001	0.75<br><.0001	0.85<br><.0001	0.86<br><.0001	0.88<br><.0001	0.92<br><.0001	1								
**48**	0.27<br>0.01	0.26<br>0.01	0.30<br>0.00	0.38<br><.0001	0.43<br><.0001	0.50<br><.0001	0.55<br><.0001	0.56<br><.0001	0.59<br><.0001	0.69<br><.0001	0.74<br><.0001	0.69<br><.0001	0.77<br><.0001	0.85<br><.0001	0.86<br><.0001	0.86<br><.0001	0.89<br><.0001	1							
**51**	0.19<br>0.07	0.33<br>0.00	0.33<br>0.00	0.44<br><.0001	0.48<br><.0001	0.47<br><.0001	0.60<br><.0001	0.59<br><.0001	0.56<br><.0001	0.68<br><.0001	0.75<br><.0001	0.74<br><.0001	0.78<br><.0001	0.86<br><.0001	0.89<br><.0001	0.91<br><.0001	0.89<br><.0001	0.91<br><.0001	1						
**54**	0.20<br>0.06	0.22<br>0.02	0.21<br>0.03	0.44<br><.0001	0.46<br><.0001	0.42<br><.0001	0.53<br><.0001	0.62<br><.0001	0.57<br><.0001	0.61<br><.0001	0.67<br><.0001	0.76<br><.0001	0.80<br><.0001	0.78<br><.0001	0.81<br><.0001	0.88<br><.0001	0.88<br><.0001	0.84<br><.0001	0.91<br><.0001	1					

Table S4, cont. Correlation coefficients and p-values for the relationship among lead levels from pre- and post-natal period, age 1-78 months

**Age Pb measured (months)**	**Pre-natal**	**1**	**3**	**6**	**9**	**12**	**15**	**18**	**21**	**24**	**27**	**30**	**33**	**36**	**39**	**42**	**45**	**48**	**51**	**54**	**57**	**60**	**66**	**72**	**78**
**57**	0.21<br>0.04	0.30<br>0.00	0.25<br>0.01	0.41<br><.0001	0.45<br><.0001	0.40<br><.0001	0.48<br><.0001	0.56<br><.0001	0.56<br><.0001	0.61<br><.0001	0.65<br><.0001	0.71<br><.0001	0.77<br><.0001	0.77<br><.0001	0.79<br><.0001	0.84<br><.0001	0.87<br><.0001	0.84<br><.0001	0.89<br><.0001	0.94<br><.0001	1				
**60**	0.26<br>0.01	0.27<br>0.01	0.30<br>0.00	0.39<br><.0001	0.45<br><.0001	0.44<br><.0001	0.54<br><.0001	0.55<br><.0001	0.56<br><.0001	0.63<br><.0001	0.67<br><.0001	0.68<br><.0001	0.73<br><.0001	0.78<br><.0001	0.80<br><.0001	0.82<br><.0001	0.82<br><.0001	0.84<br><.0001	0.88<br><.0001	0.90<br><.0001	0.90<br><.0001	1			
**66**	0.20<br>0.06	0.23<br>0.02	0.22<br>0.02	0.45<br><.0001	0.48<br><.0001	0.45<br><.0001	0.53<br><.0001	0.58<br><.0001	0.56<br><.0001	0.61<br><.0001	0.65<br><.0001	0.70<br><.0001	0.70<br><.0001	0.73<br><.0001	0.76<br><.0001	0.82<br><.0001	0.81<br><.0001	0.77<br><.0001	0.86<br><.0001	0.89<br><.0001	0.86<br><.0001	0.86<br><.0001	1		
**72**	0.19<br>0.08	0.29<br>0.00	0.27<br>0.01	0.44<br><.0001	0.43<br><.0001	0.46<br><.0001	0.56<br><.0001	0.58<br><.0001	0.51<br><.0001	0.62<br><.0001	0.69<br><.0001	0.67<br><.0001	0.66<br><.0001	0.72<br><.0001	0.75<br><.0001	0.75<br><.0001	0.77<br><.0001	0.80<br><.0001	0.84<br><.0001	0.85<br><.0001	0.83<br><.0001	0.86<br><.0001	0.89<br><.0001	1	
**78**	0.25<br>0.02	0.19<br>0.06	0.17<br>0.08	0.52<br><.0001	0.47<br><.0001	0.46<br><.0001	0.46<br><.0001	0.58<br><.0001	0.58<br><.0001	0.57<br><.0001	0.64<br><.0001	0.66<br><.0001	0.72<br><.0001	0.67<br><.0001	0.70<br><.0001	0.77<br><.0001	0.78<br><.0001	0.73<br><.0001	0.78<br><.0001	0.87<br><.0001	0.79<br><.0001	0.81<br><.0001	0.85<br><.0001	0.86<br><.0001	1

Table S5. [a,b]Regression coefficients for the an adjusted association between maximum lead exposure and *PEG3, IGF2/H19*, and *PLAGL1 /HYMAI* DMR methylation

DMR	*Both sexes	Males	Females
	coef (95%CI), p-value	coef (95%CI), p-value	coef (95%CI), p-value
PEG3			
Early childhood lead exposure (age 3 to 30 months)	-0.0007 (-0.0012, -0.0002), 0.004	-0.0014 (-0.0022, -0.0006), 0.001	-0.0003 (-0.0009, 0.0002), 0.3
Middle childhood lead exposure (age 33 to 78 months)	-0.0009 (-0.0015, -0.0004), 0.002	-0.0012 (-.0021, -0.0002), 0.02	-0.0007 (-0.0015, 0.0001), 0.08
Maximum lifetime lead exposure (birth to 78 months)	-0.0007 (-0.0012, -0.0003), 0.003	-0.0013 (-0.0021, -0.0006), 0.001	-0.0004 (-0.0010, 0.0002), 0.3
IGF2/H19			
Early childhood lead exposure (age 3 to 30 months)	-0.0004 (-0.0010, 0.0001), 0.1	-0.0001 (-0.0011, 0.0010), 0.9	-0.0005 (-0.0012, 0.0001), 0.1
Middle childhood lead exposure (age 33 to 78 months)	-0.0008 (-0.0015, -0.0002), 0.02	-0.0006 (-0.0018, 0.0005), 0.3	-0.0009 (-0.0017, -0.0001), 0.04
Maximum lifetime lead exposure (birth to 78 months)	-0.0005 (-0.0010, 0.0001), 0.1	-0.0002 (-0.0012, 0.0009), 0.8	-0.0006 (-.0012, 0.0001), 0.1
PLAGL1 /HYMAI			
Early childhood lead exposure (age 3 to 30 months)	0.0012 (-0.0007, 0.0032), 0.2	0.0017 (-0.0021, 0.0054), 0.4	0.0010 (-0.0013, 0.0033), 0.4
Middle childhood lead exposure (age 33 to 78 months)	0.0007 (-0.0018, 0.0031), 0.6	-0.0006 (-0.0048, 0.0036), 0.8	0.0005 (-0.0025, 0.0036), 0.7
Maximum lifetime lead exposure (birth to 78 months)	0.0010 (-0.0010, 0.0030), 0.4	0.0006 (-.0030, 0.0043), 0.7	0.0009 (-0.0015, 0.0033), 0.5

[a] Unstandardized regression coefficients. All models adjusted for batch (first or second) and maternal cigarette smoking (none, one half, 1 and 2 packs a day). Models of combined estimates for males and females are also adjusted for sex.

[b] Maximum lead levels for each developmental period (early childhood) were derived by arraying and identifying the maximum lead level for the relevant developmental period.
